# Protein p16 as a marker of dysplastic and neoplastic alterations in cervical epithelial cells

**DOI:** 10.1186/1471-2407-4-58

**Published:** 2004-08-31

**Authors:** Galina Volgareva, Larisa Zavalishina, Yulia Andreeva, Georgy Frank, Ella Krutikova, Darya Golovina, Alexander Bliev, Dimitry Spitkovsky, Valeriya Ermilova, Fjodor Kisseljov

**Affiliations:** 1 Institute of Carcinogenesis, N.N.Blokhin Cancer Research Center, Russian Academy of Medical Sciences, Moscow 115478, Russia; 2 P.A.Gertzen Institute of Oncology, Russian Ministry of Health, Moscow 125284, Russia; 3 Chair of Obstetrics and Gynecology, Ukrainian Medical Stomatological Academy, Poltava, Ukraine; 4 Kourion Therapeutics AG, Langenfeld, Germany; 5 Institute of Clinical Oncology, N.N.Blokhin Cancer Research Center, Russian Academy of Medical Sciences, Moscow 115478, Russia

## Abstract

**Background:**

Cervical carcinomas are second most frequent type of women cancer. Success in diagnostics of this disease is due to the use of Pap-test (cytological smear analysis). However Pap-test gives significant portion of both false-positive and false-negative conclusions. Amendments of the diagnostic procedure are desirable. Aetiological role of papillomaviruses in cervical cancer is established while the role of cellular gene alterations in the course of tumor progression is less clear. Several research groups including us have recently named the protein p16^INK4a ^as a possible diagnostic marker of cervical cancer. To evaluate whether the specificity of p16^INK4a ^expression in dysplastic and neoplastic cervical epithelium is sufficient for such application we undertook a broader immunochistochemical registration of this protein with a highly p16^INK4a^-specific monoclonal antibody.

**Methods:**

Paraffin-embedded samples of diagnostic biopsies and surgical materials were used. Control group included vaginal smears of healthy women and biopsy samples from patients with cervical ectopia. We examined 197 samples in total. Monoclonal antibody E6H4 (MTM Laboratories, Germany) was used.

**Results:**

In control samples we did not find any p16^INK4a^-positive cells. Overexpression of p16^INK4a ^was detected in samples of cervical dysplasia (CINs) and carcinomas. The portion of p16^INK4a^-positive samples increased in the row: CIN I – CIN II – CIN III – invasive carcinoma. For all stages the samples were found to be heterogeneous with respect to p16^INK4a^-expression. Every third of CINs III and one invasive squamous cell carcinoma (out of 21 analyzed) were negative.

**Conclusions:**

Overexpression of the protein p16^INK4a ^is typical for dysplastic and neoplastic epithelium of cervix uteri. However p16^INK4a^-negative CINs and carcinomas do exist. All stages of CINs and carcinomas analyzed are heterogeneous with respect to p16^INK4a ^expression. So p16^INK4a^-negativity is not a sufficient reason to exclude a patient from the high risk group. As far as normal cervical epithelium is p16^INK4a^-negative and the ratio p16^INK4a^-positive/ p16^INK4a^-negative samples increases at the advanced stages application of immunohisto-/cytochemical test for p16^INK4a ^may be regarded as a supplementary test for early diagnostics of cervical cancer.

## Background

Cervical cancer makes up about 10–12% of total women cancers [1,2] with the level of mortality in Russian population 5.0 per 100000 [1]. The tendency is being observed for the past decades towards reduction of both incidence and mortality. It is mainly due to the population-wide screening protocols in developed countries which allow identifying early asymptomatic forms of cervical carcinomas. However some problems remain to be solved concerning early detection of this type of cancer.

The main screening test for cervical cancer is the cytological smear staining technique developed by G. Papanicolaou [3] and known as Pap test. Despite evident success this test gives a substantial rate of both false-positive and false-negative results.

Histological analysis of a biopsy sample, more laborious in preparation and study as compared with that of a cytological smear, is also not absolutely efficient owing to a substantial rate of interobserver discrepancies among expert pathologists examining the same material [4].

Infection with human papilloma viruses (HPV) belonging to so-called high-risk group is the main risk factor of cervical cancer incidence [2]. To detect high risk HPVs in epithelial cells of a patient polymerase chain reaction (PCR) has been applied during the past two decades [5]. However this highly sensitive technique cannot resolve the problem of early cervical carcinoma detection also so far as many early stage lesions regress and epithelial dysplasia (i.e. cervical intraepithelial neoplasms, CINs) and carcinomas appear only in a minor part of the persons in whose epithelium (on a smear) high risk HPVs had been detected [2].

For the recent years several research groups [4,6-16] including us [11] have dwelt on the protein p16^INK4a ^for a possible supplementary marker of dysplastic and neoplastic cervical epithelium lesions. This protein belongs to the group of cyclin-dependent kinase Cdk4/6 inhibitors [17] and is encoded by tumor suppressor gene *INK 4a *(synonyms: *MTS 1*, *CDKN2*, *INK4a/ARF*). Gene *INK4a *plays an important role in the regulatory pathway Cdk-Rb-E2F. The product of this gene p16^INK4a ^prevents pRb phosphorylation by inactivating Cdk4/6; pRb keeps on binding E2F transcription factors and as a result cells stay in G1 phase not passing to DNA replication. In various tumor types *INK4a *as a bona fide tumor suppressor undergoes homozygous deletions, is inactivated by point mutations, LOH or hypermethylation; p16^INK4a ^expression is reduced or ceases under such conditions or the protein function may be impaired [18].

Peculiarity of cervical carcinomas is due to the ability of HPV oncoprotein E7 to interact with pRb and inactivate it [19]. As a result, the regulatory pathway Cdk-Rb-E2F is disrupted and the status of gene suppressor *INK4a *and its protein becomes of no importance for a cell so far as they function upstream of the site of breakage. Cells with thus inactivated pRb pass cell cycle checkpoint G1/S without any obstacle.

Reciprocallity between status of pRb and that of p16^INK4a ^commonly found in human permanent cell lines (including cervical cell line cultures) [17,20,21] as well as in primary tumor cells [9,22,23] served for a logical prerequisite of utilizing p16^INK4a ^protein as a marker of premalignant and malignant cervical epithelium cells. Functionally active gene *RB *was shown to be able to negatively regulate the expression of *INK4a *on a transcriptional level, but details of this negative feed-back loop remain obscure [24].

To estimate the applicability of p16^INK4a ^as a marker of dysplastic and neoplastic alterations in cervical epithelium cells we analyzed the expression of this protein utilizing a highly p16^INK4a ^– specific monoclonal antibody. We examined 197 samples in total. The materials studied included: 1) samples of normal epithelium of healthy women (cytological vaginal smears), 2) diagnostic biopsy samples from patients with cervical ectopia, 3) samples of CINs of various stages, 4) samples of invasive cervical cancer, 5) samples from different normal utery body and cervix tissues from women with gynecological diseases not associated with dysplastic lesions of epithelium (diagnostic biopsies and surgical materials) and 6) samples of cells from 3 cervical carcinoma cell lines. We also compare our data with the results presented by other groups working in similar directions [4,6-10,12-16].

We demonstrate in the present work that overexpression of the protein p16^INK4a ^is typical for some samples of dysplastic and neoplastic epithelium of cervix uteri. We have not found at least a single sample overexpressing p16^INK4a ^among control samples. The portion of p16^INK4a^-positive samples increases in the following row: CIN I – CIN II – CIN III – invasive carcinoma. However p16^INK4a^-negative CINs and carcinomas have also been found. All stages of CINs and carcinomas analyzed turn out to be heterogeneous with respect to p16^INK4a ^expression: side by side with the samples which express p16^INK4a ^in 25% of cells or more we detect samples which are stained poorly or lack any staining. So p16^INK4a^-negativity does not seem to be a sufficient reason to exclude a patient from the high risk group when results of Pap-test, HPV detection by PCR or histological investigation warn about possibility of poor prognosis. As far as normal cervical epithelium is p16^INK4a^-negative and the ratio p16^INK4a^-positive/ p16^INK4a^-negative samples increases at the advanced stages of CINs and carcinomas application of immunohisto-/cytochemical test for p16^INK4a ^may be regarded as a supplementary (optional) test for early diagnostics of cervical cancer.

## Methods

**Figure 1:**
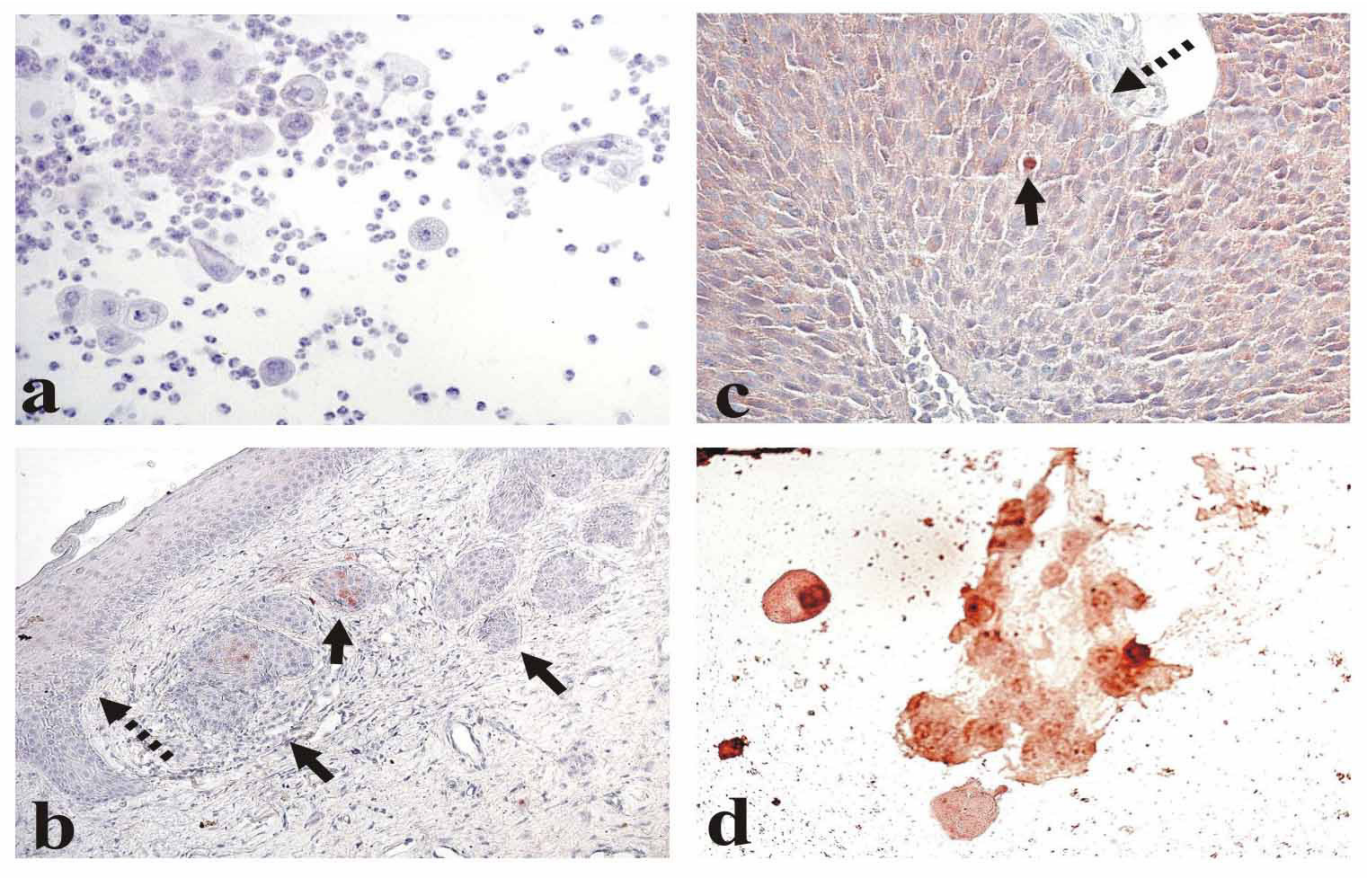
Different types of cervical cell staining with the p16^INK4a^-specific antibodies. a. Normal epithelium (smear): negative staining. b. CIN I (indicated with a dotted arrow) and cancer in situ (solid arrows): a very poor cytoplasmic staining in separate CIN I cells and sporadic staining in cancer in situ. c. Invasive squamous cell carcinoma. Diffuse cytoplasmic staining with the sole cell expressing p16^INK4a ^in the nucleus (solid arrow). The boundary with adjoining normal tissue is marked with a dotted arrow. d. HT3 cells (smear) with both nuclear and cytoplasmic subcellular location of the positive staining.

Immunohisto-/immunocytochemical study was performed on 197 samples in total. Those included 6 samples from normal epithelium of healthy women (cytological vaginal smears taken during regular examination), as well as the following surgical and diagnostic biopsy materials: 37 samples from patients with cervical ectopia, 113 samples of CINs of different stages including cancer in situ, 26 samples of invasive cervical cancer (21 squamous cell carcinomas and 5 adenocarcinomas), 12 samples of normal tissues from uterus body (myometrium) and cervix from patients with different gynecological diseases, 3 cervical cell line samples taken for positive control (see below). Apart from 9 smears (which included 6 samples from healthy women and 3 samples taken from cell cultures which served as controls) the rest 188 materials were paraffin-embedded histological blocks. The quality of smears turned out to be satisfactory for immunochemical analysis (Fig. 1a and 1d).

Neutral formalin-fixed paraffin embedded samples of biopsies and surgery materials were received from archives of N.N.Blokhin Cancer Research Center (Moscow), P.A. Gertzen Institute of Oncology (Moscow), Central Clinical Hospital (Moscow) and Fourth Clinical Hospital (Poltava, Ukraine).

For immunohistochemical analysis 4–5 μm serial sections were transferred on Histobond slides with adhesive layer (SMT Geraetehandel GmbH, Germany). The first section was stained with hematoxylin-eosin for traditional morphological analysis and verification of diagnosis by not less than two independent pathomorphologists.

Presence of high risk HPV in cervical CIN and carcinoma cells was verified by PCR [25]. Some of the squamous cell carcinomas were tested by Southern blot hybridization in addition to PCR. The results of both methods coincided completely. To detect high-risk HPV in adenocarcinomas Hybrid Capture 2 test (enabling to detect but not to discern HPV 16 and HPV 18) was used. The following portions of samples (out of those studied immunochemically) were analysed for high-risk HPV genetic material: CINs I – 9/51, CINs II – 9/32, CINs III – 19/24, invasive squamous cell carcinomas – 21/21, adenocarcinomas – 5/5. This study did not cover all CINs which we studied immunochemically due to the tiny size of most of those samples.

Vaginal normal epithelium smears obtained during regular examination from healthy women were transferred on HistoBond slides. Absence of abnormal cells was confirmed in Cytology Department of Cancer Research Center on a parallel slide. Smears were fixed for 5 min in 10% formaldehyde, washed with flowing water, and processed for further development as described for deparaffinized histological slices [9]. Cells of cervical cell lines were cultured under standard conditions in DMEM supplemented with 10% of foetal bovine serum. For immunocytochemistry cells were taken during culture receeding, dropped in a culture medium on a Histobond slide, air-dried and then processed identically to smears from healthy women.

Immunohistochemical staining was carried out using p16^INK4a^-specific monoclonal antibodies E6H4 (MTM Laboratories AG, Germany) according to protocol by Klaes et al [9].

### Controls in the course of immunohistochemical studies were as follows

#### Positive controls

1). In the beginning of the study as a whole cells of three cervical cell lines were stained first. They were SiHa (HPV16-positive), C33a and HT-3 (both HPV-negative); all of them had been characterized as expressing the protein p16^INK4a ^in earlier immunocytochemical studies with the same antibody [9]. 2). One HT-3 cell slide was stained in parallels with the first series of biopsy materials. 3). Every next group of slides intended for staining compulsorily included the slide with one of the serial sections of the CINIII or invasive carcinoma sample which had been characterized as a p16^INK4a^-positive one in our previous studies (on preceeding cuts). The analysis of the given slide series was carried out only if the positivity of the control sample was confirmed. Thus neither slide series in the present study was completely p16^INK4a^-negative.

#### Negative controls

1). One more serial cut made from p16^INK4a^-expressing material was included into every slide series meant for staining (as in variant 3 of positive controls, – see above) which was processed in a usual way but PBS was applied instead of p16^INK4a^-specific antibody. The results were regarded as valid if this slide was negative. 2). As an intrinsic negative control served adjacent to CIN or carcinoma normal tissues including stromal elements. In neither case did we find any staining in these tissues (fig. 1c).

**Figure 2:**
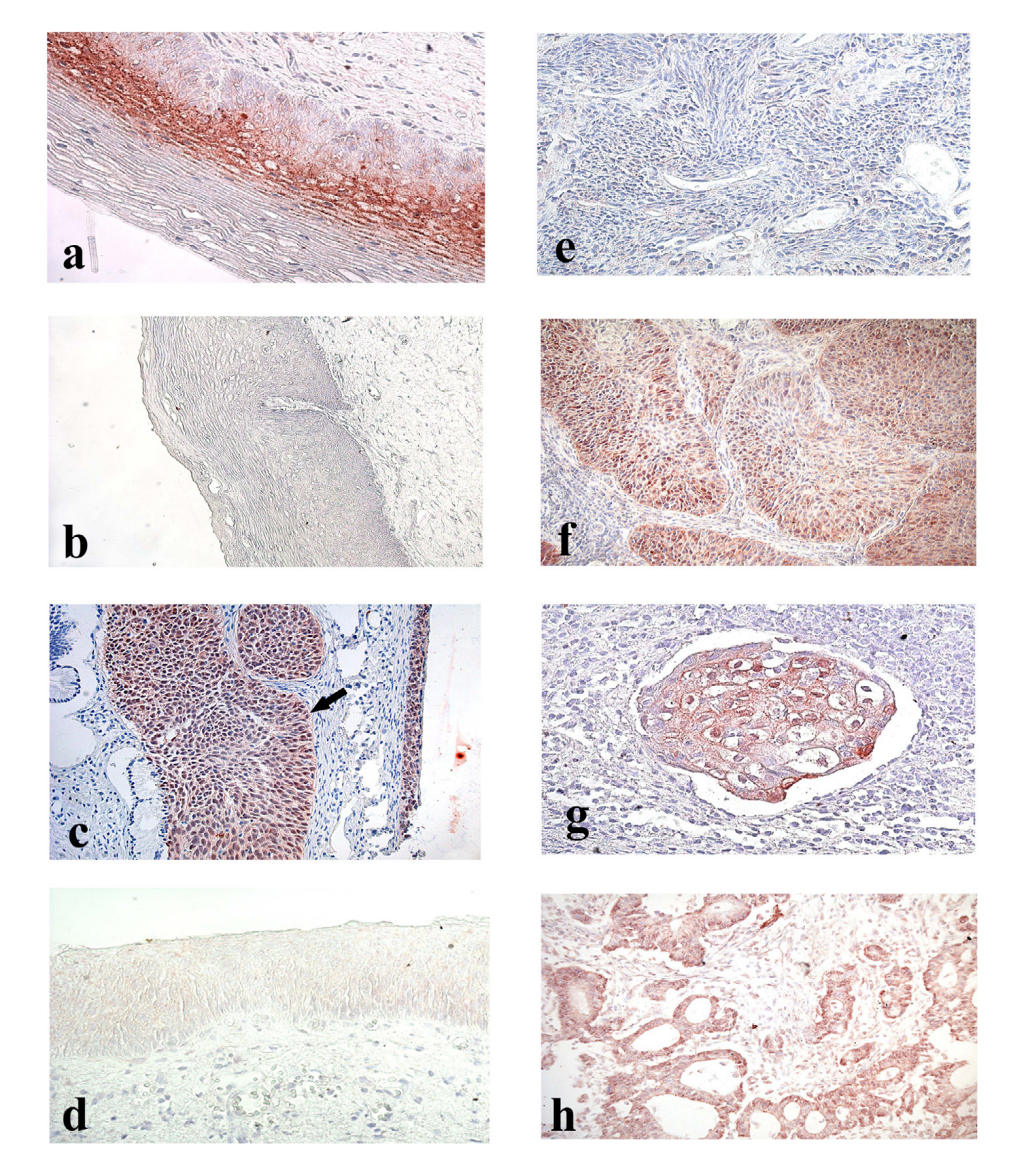
Cervical tissue samples after immunohistochemical staining with p16^INK4a^-specific antibodies. a. CIN I. Focal staining. b. CIN II. Negative staining. c. Cancer in situ (indicated with an arrow) and CIN III. Diffuse staining. d. CIN III. Negative staining. e. Squamous cell carcinoma. Negative staining. f. Squamous cell carcinoma. Diffuse staining. g. Squamous cell carcinoma embol. Diffuse staining. h. Adenocarcinoma. Diffuse staining.

In addition to four categories of staining defined by Klaes et al. [9],- poor (less than 1% of stained cells,- figure 1b), sporadic (1–5% of stained cells, – figure 1b), focal (cell clusters were stained but not more than 25% of cells were positive, – figure 2a) and diffuse (more than 25% of cells were stained, – figures 1c and 2c,2f,2g,2h), we formed one more group (negative) of those samples which totally lacked any stained cells (Figures 1a, 2b,2d and 2e). We regarded those cells as stained in which p16^INK4a ^was expressed in nuclei and/or in a cytoplasm.

## Results

Various types of p16^INK4a^-specific staining of cervix uteri normal, dysplastic and cancer cells are presented in Fig. 1a,1b,1c,1d and Fig. 2a,2b,2c,2d,2e,2f,2g,2h.

We did not register any staining in either of 6 smear samples taken from healthy women (Fig. 1a). An example of a very poor cytoplasmic staining in some separate CIN I cells with a more pronounced (sporadic, both nuclear and cytoplasmic) staining in the adjacent cancer in situ cells is shown in Fig. 1b. In one invasive carcinoma we detected cytoplasmic staining in the predominant majority of cancer cells while but sole nuclei turned out to be stained (Fig. 1c); the boundary between cancer and normal tissues coincided with the line at which the staining discontinued. As to the cervical cells cultivated in vitro taken for positive controls, in HT-3 cells the specific staining was strongly manifested both in nuclei and in a cytoplasm (Fig. 1d) while in SiHa and C33a cells it was exclusively cytoplasmic. It is not clear yet why in one and the same cervical cancer sample the protein p16^INK4a ^(normally showing its activity in nuclei) may be detected but in a cytoplasm in the majority of cells while in a number of cells both in nuclei and cytoplasm, why in some cervical cell cultures it is found solely in cytoplasm and in other cervical cell lines – in nuclei also. We did not find at least a single sample with an exceptionally nuclear staining; similar were the results by other investigators [9]. With keeping in mind that subcellular location of p16^INK4a^-specific staining varies to such a degree we scored as positive every sample in which the staining was expressed either in a cytoplasm or in both nuclei and cytoplasm.

The data of the analysis of the cervical epithelium preparations stained with monoclonal antibodies E6H4 are summarized in Table 1.

In all 37 samples from patients with cervical ectopia p16^INK4a^-positive cells were observed with low frequencies: 34 samples were negative and 3 ones (8,1%) were stained poorly.

In the group of CINs I 63% of the samples were p16^INK4a^-negative. In 7 samples (about 14%) sporadic or focal (Fig. 2a) type of staining was observed.

Group of CINs II turned out to be highly heterogeneous in terms of the ratio of p16^INK4a^-positive cells as well. Most of these samples (68%, 26 out of 38, Fig. 2b) did not differ from the samples of normal epithelium, but the rest 12 samples we attributed to poor, sporadic or focal types. In neither sample of CIN I or CIN II did we observe diffuse staining for p16^INK4a^.

Among the samples of CIN III about 54% (13 out of 24) were attributed to sporadic (Fig 1b), focal or diffuse (Fig. 2c) types. However every third CIN III sample was found to be p16^INK4a^-negative (Fig. 2d).

As to invasive cancers, only 1 out of 26 samples (3.8%) lacked p16^INK4a^-positive cells (Fig. 2e). One sample expressed the marker poorly. In 24 cases sporadic, focal or diffuse staining was observed. Diffuse staining was registered in about 50% of these samples (Figs. 1c, 2f,2g,2h). Among 14 samples of invasive carcinomas which had been stored in paraffin blocks for 7 years 1 (7.1%) turned out to be p16^INK4a^-negative, 1 (7.1%) stained poorly, 3 (21.4%) stained sporadically, 4 (28.4%)-focally and 5 (35.8%) expressed diffuse staining.

We have examined several cases with more than one type of lesion on the same slide. Examples are presented on Figures 1b and 2c. As a rule in combined cases p16^INK4a ^expression was more pronounced in cells belonging to a more advanced lesion (Fig. 1b).

All the HPV-tested samples of CINs and invasive squamous cell carcinomas were high-risk HPV-positive (data on CINs I and CINs II are shown in the additional file, results with CINs III and squamous cell carcinomas – in Table 2). High-risk HPVs were also found in all the samples of adenocarcinomas.

In reference group composed of samples from normal uterus body and cervix uteri tissues obtained from patients with gynecological diseases (stromal and glandular tissues of the cervix from patients with cervical ectopia from which squamous cell epithelium had been fully cut off; myometrium of uterus body from the patient after surgery for cervical carcinoma) the results of staining with p16^INK4a^-specific antibodies were negative in all 12 cases (Table 3).

## Discussion

Immunochemical detection of p16^INK4a ^by monoclonal antibodies shows that the overwhelming majority of invasive cervical carcinoma samples differ from normal cervical epithelium of healthy women. We found no cases of sporadic, focal or diffuse staining with E6H4 antibodies among 6 vaginal smears from healthy women as well as among 37 samples from patients with cervical ectopia including those in which ectopically localized cervical epithelium koilocytes or condyloma had been registered. In addition, in 11 samples of normal glandular and stromal cervical tissues and one sample of uterus body normal myometrium tissue which were obtained from patients with gynecological disorders p16^INK4a ^was not expressed at all.

The predominant majority of invasive carcinoma samples were both high-risk HPV-positive and p16^INK4a^-expressing. However in 2 samples not expressing any p16^INK4a ^or expressing it poorly high-risk HPV DNA sequences were detected by both PCR and Southern blot hybridization. These data are in a good agreement with those by Klaes et al., who described two samples of p16^INK4a^-negative but HPV-positive cervical cancer [9]. Among CIN III samples which were stained in our experiments poorly or lacked any staining (table 2, samples 1–6) high risk HPVs were found in 6 out of 6 tested. As to CIN I and CIN II samples expressing p16^INK4a ^in less than 1% of cells (Additional file data, samples 1–5 and 10–14, respectively), we did not find any high-risk HPV-negative case in those groups either.

Thus there seem to exist dysplastic and neoplastic lesions of cervix uteri which do not overexpress the protein p16^INK4a ^but harbor high-risk HPV DNA.

We confirm the data by Klaes et al. [9] that prolonged (for 7 years in our case) preservation of cervical carcinoma samples in paraffin blocks does not preclude the material from diffuse staining.

Monoclonal antibodies E6H4 originally described by Klaes et al [9] had been tested by those authors among a number of clones of p16^INK4a^-specific monoclonal antibodies. The following commercially available clones had been taken: 1). DCS-50.1/H4-NA29 (Oncogene Research Products, Cambridge, MA), 2). 375P (Biogenex Laboratories, San Ramon, CA), 3). ZJ11 and JC8 (NeoMarkers, Freemont, CA), 4) 05–418 (Upstate Biotechnology, Lake Placid, NY) and 5). J175–405 (PharMingen, San Diego, CA). The results of the comparative experiments by Klaes at al [9] had demonstrated that clone E6H4 had turned out to be the most specific as inferred on the lowest level of unspecific staining on different tumor cell lines, both HPV-negative and positive. As in our experiments Klaes et al. [9] registered mainly cytoplasmic staining both in cells of permanent cell lines and in preneoplastic and carcinoma samples; an exclusive nuclear staining was not detected.

We observed strong p16^INK4a^-positivity (sporadic or focal staining) in a comparatively small number of CIN I and CIN II samples (13–14 %). Among CINs III the portion of such samples increased up to 54,2%, with diffuse staining in every sixth case.

The data we have obtained for CINs of all stages rather differ from the results by Klaes et al. [9]. All of CINs II and CINs III were stained as diffuse by Klaes et al. [9]. In the present study the group of CINs as a whole is much more heterogeneous. The reasons for those discrepancies are not quite clear yet. The population of Russian and Ukrainian patients whose materials were used in the present study was extremely heterogeneous with respect to age, nationality, etc. We cannot exclude that the discrepancies mentioned may be due to these factors as had been discussed earlier [26].

Nevertheless the coincidence between our results and the data by Klaes et al [9] concerning the general trend seems much more important: the increase of p16^INK4a ^expression in dysplastic and neoplastic cervical epithelium in the course of progression.

This trend is especially evident in our study when negative cases only are taken into consideration. All 6 samples were p16^INK4a^-negative in control group. Similar was the situation in the reference group: 12/12 negative. Among CINs I, CINs III and invasive carcinomas these indices made up 32/51 (63 %), 8/24 (33%) and 1/26 (4 %) respectively.

The data presented herein allow us to conclude that hyperexpression of p16^INK4a ^when detected immunohistochemically may be regarded as a marker of dysplastic and neoplastic lesions in cervical epithelium. Positive correlation between p16^INK4a ^expression and morphological stage of the disease on the same slide (when combined cases with both dysplastic and neoplastic lesions were found) also favors this inference.

In a number of recent communications immunochemical staining for p16^INK4a ^was suggested to be performed on cytological smears [9,13-15]. Cytological approach has some advantages, so far as it enables a patient to avoid surgical intervention. That is why one may expect in the nearest future immunocytochemical version for detecting p16^INK4a ^to become rather widely used not only as an addition to surgery but also for regular examinations in outpatient clinics. In this connection it seems important to estimate frequencies of a feasible false-negativity of the test.

Keeping in mind that dysplastic lesions of all stages frequently regress and do not convert into invasive cervical carcinomas [2], we summarized the data by different groups on the frequencies of p16^INK4a^-negative invasive carcinoma samples (Table 4). We found that p16^INK4a^-negative cervical adenocarcinomas had been detected with frequencies which had varied from 0% up to 42,5% while p16^INK4a^-negative squamous cell carcinomas – with frequencies that had not exceeded 10,1%. Substantial variability of the data may be due to small numbers of samples analyzed (in some of the studies), to utilization of different types of monoclonal antibodies, to different criteria used by different research groups for the results interpretation, etc.

It seems important to realize the proper place of immunocyto-/ immunohistochemical analysis of p16^INK4a ^expression among previously developed tests for early detection of cervical carcinomas. In this connection the following points deserve mentioning. A pathologist in the course of common histological investigation usually registers some morphologic features such as zones of active mitotic divisions, multipolar mitoses, vicinity of condilomas and other formations. However when performing an immunocytochemical analysis on a smear a cytologist cannot bring in a correlation with morphological structure results of staining with p16^INK4a^-specific antibodies in separate cells. According to the results of the present study about 33% of CINs III as well as about 5% of invasive squamous cell carcinomas of cervix uteri do not differ from normal cervical epithelium with respect to p16^INK4a ^expression. Thus immunochemical staining for p16^INK4a ^(in both cyto- and histochemical versions) does not seem to be the approach that can help to fully overcome absolutely all existing ambiguities of cervical cancer early diagnostics.

As to possible sources of false positivity of immunochemical detection of the protein p16^INK4a ^the study by Agoff et al [16] deserve mentioning. According to this communication in 10 out of 10 endometrial biopsy samples studied endometrial cells were positively stained with the p16^INK4a ^– specific antibody E6H4. So far as endometrial cells may occur on vaginal smears the test does not seem to enable one to fully avoid false positivity.

## Conclusions

Overexpression of the protein p16^INK4a ^encoded by tumor suppressor gene *INK4a *is a characteristic of displastic and neoplastic alterations of cervical epithelium. The portion of p16^INK4a^-positive samples increases in the following row: CIN I – CIN II – CIN III – invasive carcinoma. All stages of CINs and carcinomas analysed are heterogeneous with respect to p16^INK4a ^expression: side by side with the samples which expressed p16^INK4a ^in 25% of cells or more we detected samples which were stained poorly or lacked any staining. According to the data we present herein p16^INK4a^-negative cervical neoplasms and carcinomas do exist. Thus the lack of p16^INK4a^-positive cells in samples from different types of cervical lesions should not be regarded as a sufficient reason for excluding a patient from the high-risk group. Despite this as far as normal cervical epithelium is p16^INK4a^-negative and the ratio p16^INK4a^-positive/ p16^INK4a^-negative samples increases at the advanced stages of CINs and carcinomas application of immunohisto-/cytochemical test for p16^INK4a ^may be regarded as an additional (optional) test for early detection of precancerous lesions in cervical epithelium.

## Competing interests

none declared

## Author's contributions

F.K., D.S., G.V. and G.F. contributed to study conception and design; E.K. and A.B. delt with sample obtaining and preliminary diagnosis; Yu.A., G.F. and V.E. were independent pathologists who confirmed the diagnosis; G.V., L.Z. and D.G. performed immunohistochemical staining and analysis; A.B. carried out HPV typing; G.V. drafted the manuscript. All authors read and approved the final version.

## Pre-publication history

The pre-publication history for this paper can be accessed here:



## Supplementary Material

Additional File 1 Data on high risk HPV genome detection by PCR in CIN I and CIN II samples. the data are presented on high-risk HPV genetic material detection in 9 CIN I and 9 CIN II samples. Click here for file

## Figures and Tables

**Table 1:** p16^INK4a ^EXPRESSION IN NORMAL, DYSPLASTIC AND NEOPLASTIC EPITHELIAL CELLS OF CERVIX UTERI

		STAINING*				
		
MATERIALS	number of samples	negative	poor	sporadic	focal	diffuse
Normal epithelium (smears)	6	6 (100)	-	-	-	-
Cervical ectopia including	37	34 (91,9)	3 (8,1)	-	-	-
C.e. + coilocytosis	10	9	1	-	-	-
C.e. + condyloma	2	1	1	-	-	-
Dysplasia:						
CINs I	51	32 (62,7)	12 (23,5)	6 (11,8)	1 (2,0)	-
CINs II	38	26 (68,4)	7 (18,4)	4 (10,5)	1 (2,7)	-
CINs III including cancer in situ	24	8 (33,3)	3 (12,5)	7 (29,2)	2 (8,3)	4 (16,7)
Invasive carcinomas including squamous cell carcinomas adenocarcinomas	26	1 (3,8)	1 (3,8)	4 (15,5)	7 (26,9)	13 (50,0)
Squamous cell carcinomas	21	1 (4,8)	1 (4,8)	3 (14,3)	6 (28,5)	10 (47,6)
Adenocarcinomas	5	-	-	1 (20,0)	1 (20,0)	3 (60,0)

* percent is given in parenthesis

**Table 2:** Data on high risk HPV genome detection by PCR in CIN III and invasive squamous cell carcinoma samples

Sample No	Type of immunochemical staining	HPV type
**CINs III**		

1	negative	16
2	negative	18
3	negative	16
4	poor	16
5	poor	16
6	poor	16
7	sporadic	16
8	sporadic	16
9	sporadic	16
10	sporadic	16
11	sporadic	16
12	sporadic	16
13	sporadic	16
14	focal	16
15	focal	16
16	diffuse	16
17	diffuse	16
18	diffuse	18
19	diffuse	18
**invasive squamous cell carcinomas**		

20	negative	16*
21	poor	16*
22	sporadic	16*
23	sporadic	16
24	sporadic	16*
25	focal	16*
26	focal	16
27	focal	16
28	focal	18
29	focal	16
30	focal	16
31	diffuse	16
32	diffuse	16
33	diffuse	16
34	diffuse	66
35	diffuse	16
36	diffuse	16
37	diffuse	16
38	diffuse	16
39	diffuse	18
40	diffuse	16 + 18

* confirmed by Southern blotting

**Table 3:** STAINING OF THE REFERENCE MATERIALS WITH p16^INK4a^-SPECIFIC MONOCLONAL ANTIBODIES

MATERIALS	number of samples	negative	poor	sporadic	focal	diffuse
Normal tissues, including	12	12	-	-	-	-
Uterus body (myometrium)	1	1	-	-	-	-
Stromal and glandular tissues of cervix uteri	11	11	-	-	-	-

**Table 4:** THE SHARE OF p16^INK4a^-NEGATIVE SAMPLES AMONG CERVICAL CARCINOMAS (LITERATURE DATA)

Research group (reference)	The share (per cent) of p16^INK4a^-negative samples
	among adenocarcinomas:
Lu et al, 1998 (7)	17/40 (42.5%)
Sano et al, 1998 (8)	4/15 (26.7%)
Milde-Langosch et al, 2001(10)	8/58 (13,8%)
Klaes et al, 2001(9)	1/7 (14.3%)
Saqi et al, 2002 (13)	0/2 (0%)
Murphy et al, 2003 (14)	0/2 (0%)
Negri et al, 2003 (15)	0/18 (0%)
Agoff et al, 2003 (16)	2/7 (28.6 %)
Present study	0/5 (0%)
	among squamous cell carcinomas:
Wong et al, 1997 (6)	8/79 (10.1%)
Sano et al, 1998 (8)	1/39 (2.8%)
Klaes et al, 2001 (9)	1/53 (1.9%)
Sano et al, 2002 (12)	0/34 (0%)
Klaes et al, 2002 (4)	0/46 (0%)
Saqi et al, 2002 (13)	0/1 (0 %)
Murphy et al, 2003 (14)	0/8 (0%)
Agoff et al, 2003 (16)	4/46 (8.7%)
Present study	1/21 (4.8%)
